# The Success of Cancer Crowdfunding Campaigns: Project and Text Analysis

**DOI:** 10.2196/44197

**Published:** 2023-03-03

**Authors:** Xupin Zhang, Xinqi Tao, Bingxiang Ji, Renwu Wang, Silvia Sörensen

**Affiliations:** 1 Department of Information Management Faculty of Economics and Management East China Normal University Shanghai China; 2 Warner School of Education and Human Development University of Rochester Rochester, NY United States

**Keywords:** cancer, GoFundme, fundraising, emotional content, sentiment analysis, campaign features, crowdfunding, gender

## Abstract

**Background:**

Recent studies have analyzed the factors that contribute to variations in the success of crowdfunding campaigns for a specific cancer type; however, little is known about the influential factors among crowdfunding campaigns for multiple cancers.

**Objective:**

The purpose of this study was to examine the relationship between project features and the success of cancer crowdfunding campaigns and to determine whether text features affect campaign success for various cancers.

**Methods:**

Using cancer-related crowdfunding projects on the GoFundMe website, we transformed textual descriptions from the campaigns into structured data using natural language processing techniques. Next, we used penalized logistic regression and correlation analyses to examine the influence of project and text features on fundraising project outcomes. Finally, we examined the influence of campaign description sentiment on crowdfunding success using Linguistic Inquiry and Word Count software.

**Results:**

Campaigns were significantly more likely to be successful if they featured a lower target amount (Goal amount, *β*=−1.949, *z* score=−82.767, *P*<.001) for fundraising, a higher number of previous donations, agency (vs individual) organizers, project pages containing updates, and project pages containing comments from readers. The results revealed an inverted U-shaped relationship between the length of the text and the amount of funds raised. In addition, more spelling mistakes negatively affected the funds raised (Number of spelling errors, *β*=−1.068, *z* score=−38.79, *P*<.001).

**Conclusions:**

Difficult-to-treat cancers and high-mortality cancers tend to trigger empathy from potential donors, which increases the funds raised. Gender differences were observed in the effects of emotional words in the text on the amount of funds raised. For cancers that typically occur in women, links between emotional words used and the amount of funds raised were weaker than for cancers typically occurring among men.

## Introduction

### Background

Crowdfunding allows individuals to obtain donations by raising large amounts of money through social media and other web-based services [1-3]. Crowdfunding platforms bridge the gap between those requesting funds and potential investors or donors. These platforms have gained considerable attention in recent years because of their convenience, efficiency, and simplicity [3]. There are various types of crowdfunding platforms, such as Indiegogo and Kickstarter, which support reward-based investments; GoFundMe, which provides help to individuals; and Mightycause, which is a nonprofit crowdfunding platform [4].

Crowdfunding platforms received 34.4 billion by 2015 for all campaigns [5]. The number of crowdfunding platforms has also grown rapidly, and the scope of their coverage has expanded globally (eg, JD Crowdfunding in China and Ketto in India) [4].

Medical crowdfunding accounts for a large percentage of crowdfunded campaigns in the United States for several reasons: neither insurance policies nor social welfare programs fully cover people’s medical costs [6]. In addition, medical expenses such as cancer treatment are typically unaffordable, even with insurance coverage [7]. In nations with health care systems that cover health care costs only partially or not at all, a serious diagnosis, such as cancer, can lead to severe financial burden or even bankruptcy. As a result, an innovative funding mechanism for covering health care expenses has emerged: medical crowdfunding. This mechanism has the potential to provide recipients with considerable advantages, including the ability to pay for necessary medical care, avoidance of medical debt, and more family time with sick loved ones [8,9]. Therefore, faced with the enormous cost of medical care, an increasing number of Americans are turning to crowdfunding platforms for help [6]. GoFundMe is a famous platform in the United States for medical crowdfunding. One-third of the total funds raised in 2017 from medical campaigns [10] are attributable to GoFundMe. The website’s tag line in the medical category is “We’re the leader in online medical fundraising” and it hosts at least 250,000 medical fundraisers each year.

Recent studies have analyzed the factors that contribute to the variations in the success of medical crowdfunding campaigns, such as the age and gender of target audiences, the race of project founders, and the characteristics of campaign images [3,4,6,11-14] In terms of text features, Ren et al [14] stated that donors were more likely to donate to crowdfunding projects for children than for adults. Igra stated that race affected the outcome of crowdfunding and that it was more difficult for Black individuals to raise money than White individuals [6]. In addition to text features, image features have a substantial impact on crowdfunding success [4]. Kochalso [3] highlighted the significance of the interrelations between various aspects.

Many scholars have focused on crowdfunding as the main type of medical crowdfunding. Previous studies have focused on 3 topics: First, the medical conditions underlying cancer crowdfunding play a role in funding success. Studies have shown that 65.4% of cancer crowdfunding patients were in the advanced stages of cancer [15]. Those campaigns that mentioned specific cancer types, specific treatments, insurance types, or out-of-pocket costs raised significantly more money than those that did not mention these characteristics [16]. The second focus of the literature is the purpose of the campaign. Song et al [17] noted that the primary purpose was to seek financial support for high medical costs resulting from various therapies, particularly those not covered by health insurance [18]. The third topic of focus is the problems with crowdfunding. Although crowdfunding platforms may alleviate the financial burden on patients with cancer, some survivors of cancer have argued that it may bring stigma in addition to financial support [19]. Cancer crowdfunding could have broader adverse effects, including promoting racial, gender [20], and regional disparities [21] in health care.

### Objective

Although several studies have focused on specific cancer types, such as thyroid [22], kidney [23], and cervical [24] cancers, none have compared the influential factors among crowdfunding campaigns for multiple cancers. In this study, we applied text analysis to data from 92,753 English-language medical crowdfunding campaigns posted on the GoFundMe website between January 1, 2019, and July 11, 2021, to determine (1) the most influential factors on the success of a campaign, including text features, (2) the differences in success rates for different cancers, and (3) the effect of emotional words in the text of campaign descriptions on the success of campaigns for different cancers.

## Methods

### Data Collection

We scraped 156,551 medical crowdfunding campaigns for cancer on the GoFundMe website from January 1, 2019, to July 11, 2021. Our data set was assembled in several steps. First, we retained only crowdfunding campaigns with English-language campaign descriptions (including countries where English is not the official language). Next, we established a cancer lexicon based on cancer types defined by the American Society of Clinical Oncology [25] and divided the campaigns into cancer categories using a rule-based method [26]; all unidentifiable campaigns were excluded. The selected sample included 92,753 cancer campaigns, summarized in Table 1. Next, Table 2 lists the campaigns’ raw data features, such as each campaign’s ID, funds already raised, target funding amount, and information regarding the text.

**Table 1:** Summary of different cancer types.

ID	Cancer name	Counts
1	Bile duct	1070
2	Bladder cancer	1825
3	Bone cancer	4407
4	Brain tumor	9651
5	Breast cancer	24,465
6	Cervical cancer	2616
7	Colorectal cancer	1180
8	Esophageal cancer	1362
9	Kidney cancer	3216
10	Leukemia	5629
11	Liver cancer	5515
12	Lung cancer	9148
13	Lymphoma	6201
14	Melanoma	1503
15	Multiple myeloma	1242
16	Ovarian, Fallopian tube, and peritoneal cancer	3256
17	Pancreatic cancer	4191
18	Prostate cancer	2425
19	Stomach cancer	2261
20	Thyroid cancer	1590

**Table 2:** Website features definition.

ID	Primitive features	Definition
1	ID	The unique number of the project
2	Raised funds	Funds already raised by the project as of the crawl date (US $)
3	Goal amount (ln)	The target amount of funds raised by the project (US $). Because the range of values of the “Goal amount” is much larger than the other variables, logarithmic transformation was done.
4	Campaign type	Binary variable to indicate whether the project was launched by an individual or institution (Institutions=0, individuals=1)
5	Donation counts	The number of times the project has accepted donations (Different donations from the same person are counted multiple times)
6	Zip code	Postal code of the area where the project is initiated to get information about the geographic location of the project
7	HasUpdate	The binary variable indicates whether the item has been updated since it was launched (Yes=1, No=0)
8	HasComment	The binary variable indicates whether the item has been reviewed since it was launched (Yes=1, No=0)
9	Created date	Creation time of the project
10	Title	Title of the project
11	Description	Detailed description text of the project

### Text Preprocessing

#### Overview

To facilitate the experimental analysis, we first transformed all text into lower case and removed irrelevant tokens, such as numbers, symbols, and stop words. Next, we performed word segmentation, including unigrams, bigrams, and trigrams [27]; this step returned 12,684,700 n-grams (n=1, 2, 3). Then, to reduce the dimensionality of text data, we chose the first 5000 different n-grams based on term frequency and calculated the inverse of the document frequency (ie, term frequency-inverse document frequency [TF-IDF]) to represent the text features of each project. In addition to using word counts as text features, we trained 300-dimensional word vectors based on the pretraining model Global Vectors for Word Representation (GloVe) [28]. Both TF-IDF and word vectors are representations of text that convert unstructured text data into structured data to be easily incorporated into the model for regression calculation. The former is based on the bag-of-words model in natural language processing, whereas the latter is based on the vector space model, which theoretically incorporates more semantic information. We used word vectors and TF-IDF features as robustness checks for comparison with the regression results based on word counts. Table 3 outlines the inferred features based on the basic features listed in Table 2. We aimed to explore the relationships between these attributes and crowdfunding outcomes.

**Table 3:** Derived features definition.

ID	Inferred features	Definition
1	Percent funded	Represents the amount of funds raised divided by the Goal amount, (ie, what proportion of the funding goal had already been collected)
2	Funding outcome	Binary variable to indicate whether the project is successful or not (successful=1; unsuccessful=0)^a^
3	Duration	Duration of the project^b^
4	Length of campaign description	Number of words in the project’s detailed description
5	Length of campaign title	Number of words in the project title
6	Number of spelling errors	Determine the number of words with spelling errors in the project details by using Python

^a^Given the positive and negative sample ratios and the large number of sponsors who failed to reach their project goals, raising a large portion of their goal money could still considerably alleviate their financial difficulties. We defined campaigns with more than 70% funding as successful. The final number of failed campaigns was 69,208, and the number of successful campaigns was 23,545.

^b^The duration of the project was obtained by subtracting the time of the day the data were crawled from the time the project started. Some recently released campaigns have not yet raised funds because of their short release times. This may have interfered with experimental results. Combined with the usual 30-40 day cycle of medical crowdfunding campaigns, the campaigns within the last 40 days were filtered out.

#### Independent Variables

We denote features with >2 multicategorical variables as dummy variables. For example, if an unordered multicategory variable such as cancer type is directly assigned 1, 2, 3, 4, and so forth, it has a mathematically sequential relationship from smallest to largest, which is not in line with reality. Therefore, we created a dummy variable for each category.

The independent variables we used were Funds raised to date, *Goal Amount*, *Campaign Type* (Individual vs Organizational), *Donation Counts, HasUpdate, HasComment, Percent Funded to Date, Duration of Campaign, Length of Campaign Description, Length of Campaign Title, and Number of Spelling Errors*. Because the range of values of the target amount for fundraising (*Goal amount*) is much larger than that of other variables, we log transformed this variable to minimize the impact of its large range of values on the regression results. For more detailed definitions of the variables, please see Tables 2 and 3.

#### Dependent Variables

We defined *Funding outcome* (Success or Failure) as the dependent variable. We used a binary variable to indicate whether a project was successful (successful=1; unsuccessful=0). Given the positive and negative sample ratios and the large number of sponsors who failed to reach their project goals (*Goal amount*), raising a large portion of their goal money could substantially alleviate their financial difficulties. We defined campaigns with over 70% of *Percent Funded* as successful. The number of failed campaigns was 69,208 and the number of successful campaigns was 23,545.

Tables 4 and 5 present the descriptive summary information of the variables and the correlation between the variables. We eventually maintained 89,828 cancer crowdfunding campaigns after excluding the outliers.

**Table 4:** Descriptive statistics for study variables.

Variables	Values					
	Mean (SD)	Minimum	25%	50%	75%	Maximum
Funding outcome	0.199 (0.399)	0.000	0.000	0.000	0.000	1.000
Campaign type	0.978 (0.146)	0.000	1.000	1.000	1.000	1.000
Donation count	55.015 (76.114)	2.000	10.000	27.000	66.000	582.000
Length of description	141.947 (102.578)	1.000	70.000	113.000	183.000	660.000
Length of title	5.023 (1.951)	1.000	4.000	5.000	6.000	14.000
Goal amount (ln)	9.007 (1.382)	0.000	8.517	9.210	9.903	20.030
HasUpdate	0.229 (0.419)	0.000	0.000	0.000	0.000	1.000
HasComment	0.388 (0.4872)	0.000	0.000	0.000	1.000	1.000
Duration	16.007 (8.695)	1.367	8.400	16.467	23.667	30.733
Number of spelling mistakes	10.549 (9.531)	0.000	4.000	8.000	14.000	60.000
Bile duct	0.011 (0.105)	0.000	0.000	0.000	0.000	1.000
Bladder cancer	0.020 (0.138)	0.000	0.000	0.000	0.000	1.000
Bone cancer	0.047 (0.211)	0.000	0.000	0.000	0.000	1.000
Brain tumor	0.101 (0.302)	0.000	0.000	0.000	0.000	1.000
Breast cancer	0.266 (0.441)	0.000	0.000	0.000	1.000	1.000
Cervical cancer	0.028 (0.166)	0.000	0.000	0.000	0.000	1.000
Colorectal cancer	0.013 (0.112)	0.000	0.000	0.000	0.000	1.000
Esophageal cancer	0.015 (0.12)	0.000	0.000	0.000	0.000	1.000
Kidney cancer	0.035 (0.183)	0.000	0.000	0.000	0.000	1.000
Leukemia	0.060 (0.238)	0.000	0.000	0.000	0.000	1.000
Liver cancer	0.060 (0.237)	0.000	0.000	0.000	0.000	1.000
Lung cancer	0.100 (0.299)	0.000	0.000	0.000	0.000	1.000
Lymphoma	0.067 (0.25)	0.000	0.000	0.000	0.000	1.000
Melanoma	0.016 (0.126)	0.000	0.000	0.000	0.000	1.000
Multiple myeloma	0.013 (0.114)	0.000	0.000	0.000	0.000	1.000
Ovarian	0.035 (0.184)	0.000	0.000	0.000	0.000	1.000
Pancreatic cancer	0.045 (0.208)	0.000	0.000	0.000	0.000	1.000
Prostate cancer	0.026 (0.16)	0.000	0.000	0.000	0.000	1.000
Stomach cancer	0.024 (0.154)	0.000	0.000	0.000	0.000	1.000
Thyroid cancer	0.017 (0.13)	0.000	0.000	0.000	0.000	1.000

**Table 5:** Pearson correlation matrix.

ID	Variable	Value								
		2	3	4	5	6	7	8	9	10
1	Funding outcome	–0.205 (*P*<.001)	0.302 (*P*<.001)	–0.133 (*P*<.001)	–0.026 (*P*<.001)	–0.267 (*P*<.001)	0.109 (*P*<.001)	0.172 (*P*<.001)	–0.032 (*P*<.001)	–0.122 (*P*<.001)
2	Campaign type	1.00	0.046 (*P*<.001)	0.044 (*P*<.001)	–0.036 (*P*<.001)	0.194 (*P*<.001)	0.058 (*P*<.001)	0.064 (*P*<.001)	–0.010 (*P*=.002)	0.013 (*P*<.001)
3	Donation count	—^a^	1.00	0.280 (*P*<.001)	0.036 (*P*<.001)	0.403 (*P*<.001)	0.295 (*P*<.001)	0.527 (*P*<.001)	–0.107 (*P*<.001)	0.313 (*P*<.001)
4	Length of description	—	—	1.00	0.141 (*P*<.001)	0.272 (*P*<.001)	0.114 (*P*<.001)	0.142 (*P*<.001)	0.007 (*P*=.03)	0.751 (*P*<.001)
5	Length of title	—	—	—	1.00	0.066 (*P*<.001)	0.033 (*P*<.001)	–0.004 (*P*=.27)	–0.095 (*P*<.001)	0.100 (*P*<.001)
6	Goal amount (ln)	—	—	—	—	1.00	0.158 (*P*<.001)	0.235 (*P*<.001)	–0.078 (*P*<.001)	0.232 (*P*<.001)
7	HasUpdate	—	—	—	—	—	1.00	0.432 (*P*<.001)	–0.089 (*P*<.001)	0.110 (*P*<.001)
8	HasComment	—	—	—	—	—	—	1.00	–0.025 (*P*<.001)	0.150 (*P*<.001)
9	Duration	—	—	—	—	—	—	—	1.00	–0.020 (*P*<.001)
10	Number of spelling mistakes	—	—	—	—	—	—	—	—	1.00

^a^Not applicable.

### Statistical Modeling Through Penalized Logistic Regression

We used penalized logistic regression (PLR) based on the *glmnet* package [29] in R programming language to explore the relationship between each project feature and crowdfunding success. PLR adds regular optimization compared with traditional logistic regression, controlled by the parameter *α* to use L1 regularity (*α*=1; lasso regression) or L2 regularity (*α*=0; ridge regression).

The use of L1 regularity is also known as lasso regression. Feature selection can be realized by limiting the sum of the absolute values of the estimates, such that some coefficients are equal to zero. First, because of the large number and collinearity of English phrases in our sample, we used lasso regression to retain important phrase features and set the other relevant features to zero. The use of L2 regularity is otherwise referred to as ridge regression. This type of regression is similar to lasso regression in terms of partially reducing model complexity; however, the number of features does not change. Ridge regression does not cause the coefficients to equal zero but instead only minimizes them. This outcome is not conducive to feature reduction because the model remains complex when dealing with number of features. These aspects can compromise the model performance. Therefore, we adopted lasso regression to make the model more parsimonious.

Second, overfitting is a common problem in model prediction; model parameters sometimes fit the training data too closely and hinder the general prediction ability of an overall data set. We used 10-fold cross-validation to address the covariance in our data and to prevent overfitting. Finally, because a single campaign description in the data set could only contain a mere fraction of possible English phrases, a large text sparsity matrix was obtained. Lasso regression is better suited for managing sparse data.

Because the range of values of the goal amount is much larger than that of the other variables, it will adversely affect the model convergence. Therefore, logarithmic processing was performed to narrow the range of the values. The variables were then normalized. We first added 39 control variables to the baseline model and then incorporated English phrases into the model to observe changes in the model’s explanatory power. As shown in Table 6, the deviance of the model (ie, model 1: intercept only) was 105,093.11. The cross-validation (CV) error reflects the proportion of successful samples in the original data set versus the full data set (CV error=25.38%). After adding control variables (ie, model 2), the deviance dropped to 80,151.81, and the CV error dropped to 19.57%, indicating that including control variables greatly improved the model’s explanatory power. The deviance and the CV error were further reduced (deviance=62,268.85; CV error=15.56%) upon adding English phrase features (based on the count word frequency matrix). We also used other vectorized representations of English phrases based on TF-IDF and GloVe to check the robustness of the model. The results showed that the deviation of the count-based method was better than that of GloVe and slightly lower than that of TF-IDF; the CV error did not differ substantially from both, reinforcing the validity of the model (model 3).

**Table 6:** Summary of different models fits.

	Model 1	Model_2	Model 3_count	Model 4_glove	Model 5_tf-idf
Deviance	105,093.11	80,151.81	62,268.85	67,641.44	62,494.18
Degrees of freedom	92,752	92,807	97,037	93,101	97,132
CV^a^ error (%)	26.38	19.57	15.56	14.54	15.61

^a^CV: cross-validation.

## Results

The results section is divided into 3 thematic subsections: general campaign features, type of cancer, and text features.

### General Campaign Features

Table 7 shows the model regression coefficients for the predictors of success for campaign projects. Among the general characteristics, the number of donations (Donation Count, *β*=1.404, *z* score=77.935, *P*<.001), whether the project has an update record (Page Has Updates, *β*=0.283, *z* score=21.175, *P*<.001), and presence of comments (Page Has Comments, *β*=0.671, *z* score=43.279, *P*<.001) were positively associated with a project’s fundraising success. Four other campaign features had a strong negative effect on a project’s fundraising success: the type of fundraisers, with individual campaigns having less success (Campaign Type, *β*=−0.281, *z* score=−20.978, *P*<.001), a high project fundraising goal (Goal amount, *β*=−1.949, *z* score=−82.767, *P*<.001), the project’s duration (Duration of Campaign, *β*=−0.112, *z* score=−8.383, *P*<.001), and the number of misspelled words in the project text (Number of spelling errors, *β*=−1.068, *z* score=−38.79, *P*<.001).

**Figure 1:**
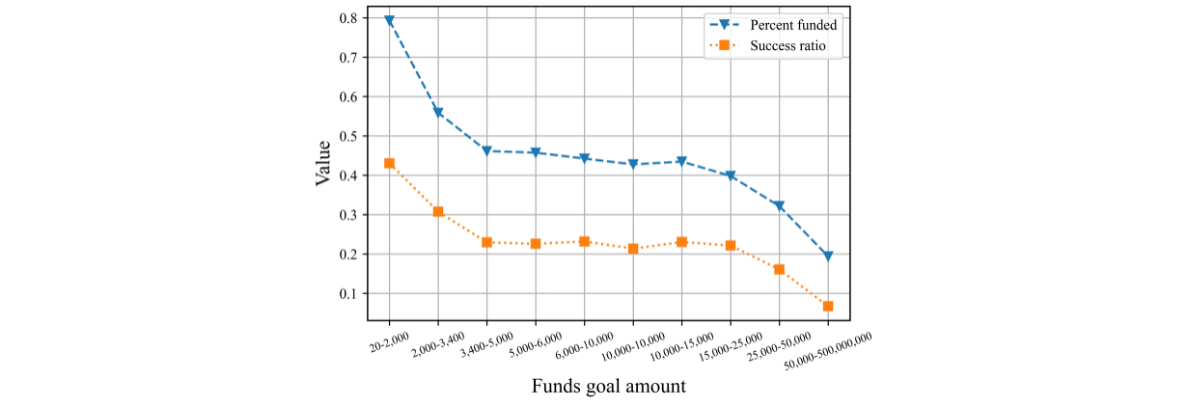
Percent funded and success rate by fundraising the target amount range.

In summary, features related to funding have a strong connection to crowdfunding success. As depicted in Figure 1, a higher-than-average success rate (percent funded) is associated with smaller campaign targets (ie, those in the US$ 1-US $1500 range). These success metrics drop off as the funding target grows but remain relatively stable between 3400 and 10,000 until further decreasing for the ultrahigh dollar campaigns (ie, more than US $ 25,000).

In contrast to fundraising targets, project duration was negatively associated with success.

In the analysis, we could not limit project timeframes because GoFundMe does not set deadlines for projects, so donations can continue after a campaign goal has been reached. Fundraising projects typically spanned 30-40 days; if organizers did not make timely fundraising progress early in their campaigns, the amount raised tended not to change substantially thereafter, even though donations continued to arrive after the first surge.

**Table 7:** Features with influential effect on success of campaign based on penalized logistic regression.

ID	Name	Coefficient	*z* score	SE	*P* value
1	Intercept	−1.781	−138.414	0.013	<.001
2	Campaign type (organization vs individual)	−0.281	−20.978	0.011	<.001
3	Donation count	1.404	77.935	0.016	<.001
4	Length of description	−0.044	−1.833	0.020	.32
5	Length of title	−0.016	−1.212	0.011	.03
6	Goal amount (ln)	−1.949	−82.767	0.020	<.001
7	PageHasUpdates	0.283	21.175	0.012	<.001
8	PageHasComments	0.671	43.279	0.013	<.001
9	Duration of campaign	−0.112	−8.383	0.011	<.001
10	Number of spelling errors	−1.068	−38.79	0.073	<.001
11	Bile duct	0.100	8.037	0.012	<.001
12	Bladder cancer	0.000	−0.020	0.015	.98
13	Bone cancer	−0.023	−1.178	0.019	.24
14	Brain tumor	−0.003	−0.125	0.025	.90
15	Breast cancer	−0.011	−0.303	0.035	.76
16	Cervical cancer	−0.049	−2.938	0.017	.003
17	Colorectal cancer	0.010	0.715	0.014	.48
18	Esophageal cancer	0.040	2.986	0.013	.003
19	Kidney cancer	−0.022	−1.224	0.018	.22
20	Leukemia	−0.012	−0.566	0.021	.57
21	Liver cancer	−0.014	−0.666	0.021	.51
22	Lung cancer	−0.070	−2.755	0.025	.006
23	Lymphoma	0.044	2.051	0.022	.04
24	Melanoma	0.025	1.809	0.014	.07
25	Multiple myeloma	0.026	1.868	0.014	.06
26	Ovarian	−0.003	−0.199	0.017	.84
27	Pancreatic cancer	0.052	2.803	0.019	.005
28	Prostate cancer	−0.008	−0.512	0.016	.61
29	Stomach cancer	−0.004	−0.228	0.016	.82
30	Thyroid cancer	N/A^a^	N/A	N/A	N/A

^a^N/A: not applicable.

### Types of Cancer

**Figure 2:**
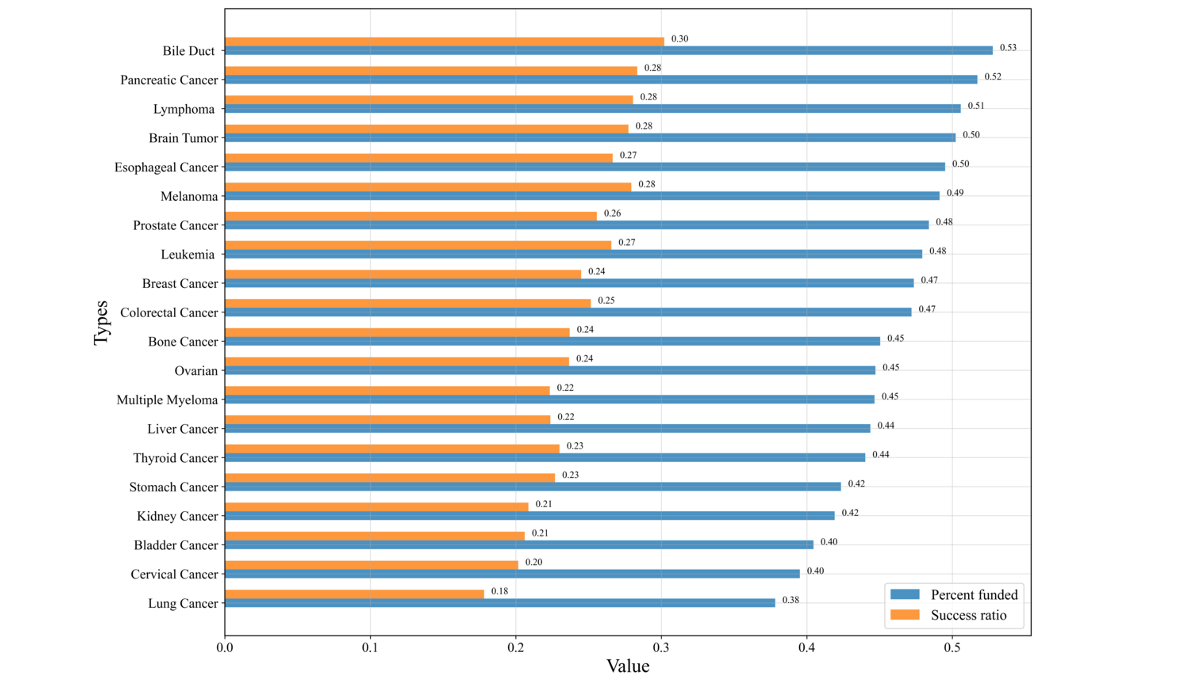
Average percent funded and success rates by cancer type.

As shown in Figure 2, we observed the fundraising performance of different cancer campaigns based on 2 indicators: the average percentage funded and the success rate. The regression coefficients indicated wide variability in project success according to the cancer type. Bile duct cancer (cholangiocarcinoma; *β*=0.10, *z* score=−8.04, *P*<.001), esophageal cancer (*β*=0.04, *z* score=−2.99, *P*=.003), melanoma (*β*=0.02, *z* score=1.81, *P*=.07), and pancreatic cancer (*β*=0.05, *z* score=2.80, *P*=.005) had a substantial positive effect on funding outcome, with the highest success rates for activities involving cholangiocarcinoma (average success ratio: 30.20% and average percent funded: 52.77%). In contrast, cervical cancer (*β*=−0.05, *z* score=−2.94, *P*=.003) and lung cancer (*β*=−0.07, *z* score=−2.76, *P*=.006) were negatively associated with funding outcomes, with lung cancer having the lowest success rate for all cancers (average success ratio: 17.82% and average percent funded: 37.83%).

### Text Features

#### Length of Text

The project text was divided into 2 parts: the project title and a detailed description. Table 8 shows that no considerable differences emerged in the campaigns’ average percent funded and success rate based on title length. Most project titles clearly stated information such as beneficiaries and disease names. In terms of text length, the headlines of most sections ranged between 4 and 7 words; this length demonstrated a slight advantage over longer or shorter headlines.

**Figure 3:**
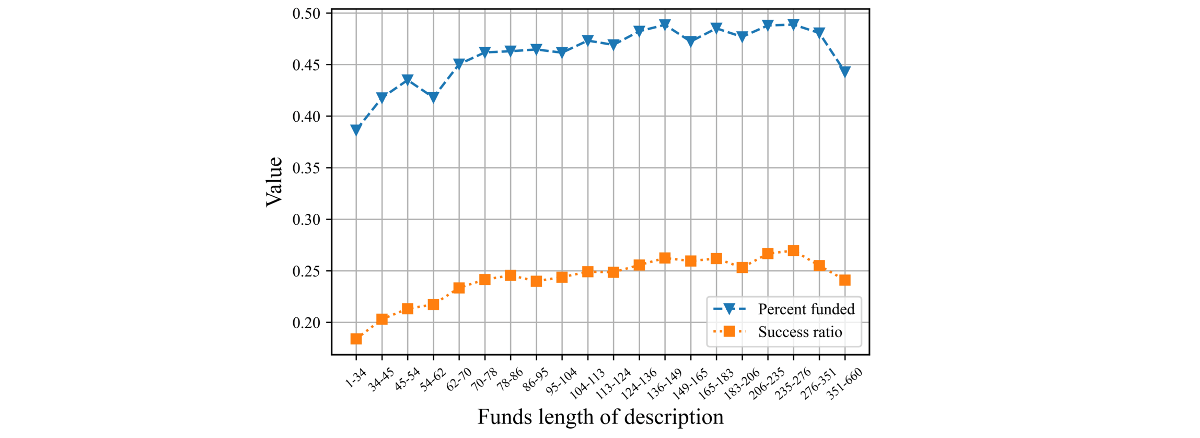
Average percent funded and success rates by length of the project description.

The relationship between project description length and fundraising results is illustrated in Figure 3. Similar to the length of the project title, the campaigns’ average percent funded and success rate initially increased with length and then showed a decreasing trend.

**Table 8:** Fundraising results in different title length intervals.

Length interval (%)	Funds current to goal (%)	Success ratio (%)
1-4 words (22.96)	41.67	24.78
4-7 words (53.74)	41.86	24.83
7-10 words (22.13)	40.45	23.26
10-14 words (1.17)	36.19	20.75

#### Content of Text

We extracted the top 50 phrases most associated with project success or failure based on the logistic regressions that had the top 50 highest or lowest *β* coefficients. The ordering of these phrases is presented through Tables 9 and 10. Most English phrases related to a project’s fundraising success were bigrams and trigrams, both of which contain more complex semantic information than unigrams. Specific medical terms such as “thyroid,” “cancer treatments,” “pancreatic cancer,” and “Covid” also played positive roles. Phrases that express gratitude (eg, “thank you,” “need your help,” and “please pray for”) and those indicating charity fundraising activities (eg, “hair,” “medical center,” and “cancer research”) were also positive predictors of success. These words and phrases were also more likely to elicit support, as will be discussed in detail below.

Among the phrases most related to project failure, many were found to relate to women (eg, “grandmother,” “aunt,” “mother,” and “she”). Medical phrases associated with project failure included terms such as “ductal,” and “cervical cancer.” Thus, women seem to be at a disadvantage compared to men in terms of raising money via crowdfunding. Structurally, most phrases were unigrams and bigrams; that is, they tended to be simpler and conveyed less semantic information. Finally, compared with words common in successful campaigns, failed campaigns featured more pessimistic words such as “failure,” “cannot,” and “negative.”

**Table 9:** Top 50 phrases signaling that the project will be funded.

Rank	Phrases	*β*
1	Hair	2.32551
2	in_aid_of	1.873002
3	thank_you	1.834425
4	Rare	1.827049
5	need_your_help	1.628172
6	the_surgeon	1.57181
7	trip	1.513046
8	lucky	1.498507
9	charity	1.459997
10	pray	1.365733
11	guy	1.35132
12	dog	1.330811
13	thyroid	1.329916
14	gift	1.273839
15	please_pray_for	1.263614
16	cancer_treatments	1.260775
17	teacher	1.234377
18	social_media	1.220046
19	staff	1.211656
20	june	1.196617
21	medical_center	1.172205
22	and_thank_you	1.169583
23	pancreatic_cancer	1.16822
24	bit	1.166253
25	radiation	1.161074
26	money_in	1.1278221
27	houston	1.1093524
28	cancer_research	1.1046241
29	her_son	1.1004744
30	you_know	1.086452
31	remove_the_tumor	1.0801651
32	meals	1.0658478
33	more_information_about	1.0629098
34	covid	1.0564797
35	and_thank_you	1.0365421
36	miles	1.0346791
37	for_weeks	1.0330821
38	college	1.0254821
39	hospice	1.0169092
40	term	1.0140427
41	friends	1.0130013
42	drug	1.0063572
43	the_lord	1.0060987
44	summer	0.9905393
45	to_recover	0.986438
46	any_way	0.984336
47	health_insurance	0.9731374
48	year_old_son	0.9702704
49	the_world	0.9631651
50	Goal	0.9533642

**Table 10:** Funding failure phrases.

Rank	Phrases	*β*
1	Ductal	–3.12579
2	cervical cancer	–3.06354
3	Had	–3.0504
4	Grandmother	–3.03949
5	Ductal	–2.396
6	Weight	–2.35016
7	Save	–2.23608
8	UK	–2.15523
9	Healing	–1.84889
10	Give	–1.81071
11	Daughter	–1.76682
12	Grandfather	–1.74575
13	Conditions	–1.73582
14	and from	–1.65317
15	Aunt	–1.64155
16	Hospitals	–1.56082
17	Mother	–1.55073
18	Progress	–1.53249
19	Problems	–1.51119
20	Life	–1.47464
21	Leg	–1.4316
22	Still	–1.33643
23	those who	–1.3125
24	University	–1.28943
25	Failure	–1.27174
26	Eating	–1.26668
27	can not	–1.26453
28	She	–1.21934
29	high school	–1.21249
30	Dream	–1.21205
31	Account	–1.20582
32	Medications	–1.20352
33	Health	–1.17978
34	disease and	–1.16989
35	Society	–1.15781
36	of the	–1.15107
37	World	–1.13135
38	Alive	–1.10527
39	Little	–1.10163
40	Dr	–1.08841
41	told that	–1.08782
42	Afford	–1.0869
43	Count	–1.07107
44	Advised	–1.06985
45	Bills	–1.06203
46	living in	–1.04171
47	Father	–1.03768
48	a donation	–1.03597
49	Breathing	–1.03458
50	Negative	–1.02679

### Linguistic Inquiry and Word Count–Based Sentiment Analysis

The social and psychological tone or sentiment of a GoFundMe campaign may also impact its success. Research suggests that the Linguistic Inquiry and Word Count (LIWC) software is ideal for identifying emotions in language [30]. In addition, LIWC can reveal more than just the emotions underlying it, it can even identify the social status, motives, and gender of the respondent [31]. The software can uncover individuals’ emotions, thinking styles, and social concerns [30]. Therefore, we used LIWC to capture the fundraiser’s emotional, cognitive, and structural components present in the GoFundMe campaigns’ descriptions. Using LIWC version 2022, we focused on 22-word categories [13], including affective, social, cognitive, perceptual, biological, drive-related, and personal words.

In *step one* of the LIWC analysis, we conducted sentiment analysis of the project descriptions. The software returned scores for 22 categories of words that occurred frequently in campaigns for the 20 cancer categories. In *step 2*, we performed a correlation analysis between the number of successful campaigns and the 5-year survival rate for the campaigns for cancers with the top 25% and bottom 25% success rates, as determined by the binary success rate variable. In *step 3*, we conducted binary logistic regression on the LIWC results of 3 cancers that most often affected men (prostate cancer, colorectal cancer, and lung cancer) and women (breast cancer, ovarian cancer, and cervical cancer) to examine how different words in campaign descriptions affected the success of these projects, as shown in Table 11.

For step 1, we divided the 20 cancer categories into quartiles based on the success rate of the category and focused on the top 25% (5/20) (bile duct cancer, brain tumor, lymphoma, pancreatic cancer, and melanoma) and the bottom 25% (5/20) (thyroid cancer, bladder cancer, kidney cancer, cervical cancer, and lung cancer). The proportion of positive words used for the top 25% (5/20) of cancers was higher than for the bottom 25% (5/20); that is, campaigns in the most successful cancer campaign categories were more likely to use positive words. The bottom 25% (5/20) of cancers barely used words related to expressing one’s feelings. Focusing on certainty- and risk-related words, we found that the top 25% (5/20) of cancers with the highest average campaign success rates rarely used words related to certainty, whereas nearly one-third of the bottom 25% (5/20) of cancers did so. These words were negatively related to project success; that is, the presence of words such as “really” and “actually” in project descriptions were related to lower success rates. Campaigns with low success rates mentioned risk-related words (including danger and risk) more often and indicated a painful atmosphere, which partly reduced readers’ willingness to contribute.

Step 2, focusing on the cancers with the lowest 25% and highest 25% success rates, we conducted a correlation analysis between the number of successful campaigns and 5-year survival rate. The results are presented in Table 12. The relationship between success and survival rates for the lowest 25% of the campaigns was not significant, but for the highest 25%, it was significant (*P*<.05) and showed a negative correlation, meaning that the higher the survival rate among the most successful campaigns, the lower the success rate was likely to be. This pattern partly supports our theory that higher than 5-year survival rates are less likely to attract success in crowdfunding: higher survival rates lead campaign readers to donate less money.

In step 3, we analyzed the word sentiments of gender-linked cancers. The regression results revealed that the use of positive words in the description was positively related to crowdfunding success in breast cancer (*P*<.001), ovarian cancer (*P*<.001), colorectal cancer (*P*=.002), and lung cancer (*P*=.005). In addition, the negative emotions detected by LIWC were negatively associated with crowdfunding success in prostate cancer, colorectal cancer, and lung cancer (*P*<.01), suggesting that more negative emotions in the descriptions predicted the lower success of these campaigns. In the 2 types of female-linked cancers (breast cancer and ovarian cancer) a positive relationship between anxiety words, such as *worried* and *fearful* (*P*<.05) and funding success was found; sadness words (including *crying* and *sad*) had a positive association with crowdfunding success only for lung cancer.

Words related to friendship, such as *pal*, *buddy*, and *coworker* used in the project description contributed to the success of lung cancer crowdfunding. References to woman (including *girl, her,* and *mom*) had a negative effect on both female-linked and male-linked cancer funding success rates, suggesting that mentioning women in the campaign description may reduce success rates.

In the larger category of cognitive words, health-related words such as *cough*, *symptom*, and *hospital* had a considerable negative effect on both female-linked and male-linked cancer campaign success (*P*<.05). In contrast, ingestion words (including *hungry, hungrier,* and *hungriest*) had a considerable positive impact on the success rate of female-linked cancers, such as breast cancer (*P*<.05).

The use of words related to money (including *owe, cash,* and *pay*) and religion (including *altar* and *church*) had a considerable negative impact on the success (*P*<.05) of campaigns related to both male-linked and female-linked cancers.

**Table 11:** Linguistic Inquiry and Word Count and binary logistic regression.

		Female cancers				Male cancers		
		Cancers that most often affect women				Cancers that most often affect men		
		Breast cancer	Ovarian	Cervical cancer	Prostate cancer		Colorectal cancer	Lung cancer
Success number		6216	802	544	646		314	1699
Success rate, %		25.40	24.60	20.80	26.60		26.60	18.60
**Affective**								
	Positive emotion	0.093 (*P*<.001)	0.157 (*P*<.001)	0.068 (*P*=.17)	0.080 (*P*=.10)		0.239 (*P*=.002)	0.077 (*P*=.005)
	Negative emotion	−0.167 (*P*<.001)	−0.325 (*P*=.005)	−0.154 (*P*=.21)	−0.250 (*P*=.04)		−0.551 (*P*=.009)	−0.158 (*P*=.03)
	Anxiety words	0.093 (*P*=.09)	0.253 (*P*=.12)	0.199 (*P*=.27)	0.336 (*P*=.08)		0.501 (*P*=.09)	0.177 (*P*=.09)
	Anger words	0.249 (*P*<.001)	0.441 (*P*=.04)	0.342 (*P*=.20)	0.104 (*P*=.65)		0.145 (*P*=.76)	0.323 (*P*=.02)
	Sadness words	0.067 (*P*=.44)	0.328 (*P*=.16)	0.226 (*P*=.37)	0.254 (*P*=.34)		0.312 (*P*=.48)	0.295 (*P*=.02)
**Social**								
	Friend words	0.062 (*P*=.06)	−0.083 (*P*=.44)	0.138 (*P*=.20)	−0.152 (*P*=.18)		0.167 (*P*=.37)	0.155 (*P*=.01)
	Female references	−0.052 (*P*<.001)	−0.038 (*P*=.003)	−0.028 (*P*=.05)	−0.180 (*P*=.001)		−0.035 (*P*=.10)	−0.028 (*P*=.001)
**Cognitive**								
	Insight words	0.053 (*P*<.001)	0.117 (*P*=.001)	0.052 (*P*=.21)	0.095 (*P*=.01)		0.071 (*P*=.25)	0.063 (*P*=.005)
	Discrepancy words	−0.084 (*P*<.001)	−0.115 (*P*=.001)	−0.069 (*P*=.07)	−0.150 (*P*<.001)		−0.156 (*P*=.02)	−0.099 (*P*<.001)
	Tentative words	−0.017 (*P*=.25)	−0.032 (*P*=.47)	−0.125 (*P*=.02)	−0.143 (*P*=.002)		−0.054 (*P*=.49)	−0.066 (*P*=.02)
	Certainty words	0.000 (*P*=.99)	0.027 (*P*=.77)	−0.080 (*P*=.48)	−0.102 (*P*=.33)		0.06 (*P*=.71)	0.037 (*P*=.52)
	Differentiation	−0.086 (*P*<.001)	−0.085 (*P*=.02)	−0.049 (*P*=.25)	−0.121 (*P*=.001)		−0.003 (*P*=.96)	−0.075 (*P*=.001)
**Perceptual**								
	See words	0.059 (*P*=.02)	0.033 (*P*=.65)	0.091 (*P*=.34)	−0.200 (*P*=.02)		0.148 (*P*=.19)	0.027 (*P*=.56)
	Feel words	−0.046 (*P*=.17)	0.150 (*P*=.11)	0.115 (*P*=.31)	−0.039 (*P*=.73)		−0.039 (*P*=.83)	−0.002 (*P*=.97)
**Biological**								
	Health words	−0.072 (*P*<.001)	−0.046 (*P*=.01)	−0.069 (*P*=.005)	−0.084 (*P*<.001)		−0.067 (*P*=.04)	−0.069 (*P*<.001)
	Ingestion words	0.110 (*P*=.001)	−0.064 (*P*=.51)	0.076 (*P*=.53)	−0.065 (*P*=.46)		−0.058 (*P*=.72)	−0.125 (*P*=.06)
**Drive**								
	Reward words	0.115 (*P*=.002)	0.168 (*P*=.14)	0.012 (*P*=.94)	0.207 (*P*=.04)		0.333 (*P*=.08)	0.285 (*P*<.001)
	Risk words	−0.075 (*P*=.01)	−0.192 (*P*=.03)	−0.009 (*P*=.92)	0.032 (*P*=.72)		0.159 (*P*=.29)	−0.122 (*P*=.03)
**Personal**								
	Work words	−0.001 (*P*=.90)	0.061 (*P*=.04)	−0.005 (*P*=.89)	0.020 (*P*=.50)		−0.017 (*P*=.75)	0.043 (*P*=.03)
	Leisure words	0.143 (*P*<.001)	0.124 (*P*=.20)	0.358 (*P*=.004)	0.229 (*P*=.006)		−0.037 (*P*=.83)	0.118 (*P*=.04)
	Money words	−0.065 (*P*<.001)	−0.063 (*P*=.04)	−0.119 (*P*=.001)	−0.059 (*P*=.05)		−0.126 (*P*=.02)	−0.097 (*P*<.001)
	Religion words	−0.309 (*P*<.001)	−0.376 (*P*<.001)	−0.353 (*P*<.001)	−0.314 (*P*<.001)		−0.183 (*P*=.02)	−0.232 (*P*<.001)

**Table 12:** Correlation between funding success number and 5-year survival rate (the top 25% and the bottom 25%).

		Success number (top 25%)	5-year survival rate (top 25%)	Success number (bottom 25%)	5-year survival rate (bottom 25%)
**Success number (top 25%)**					
	Pearson correlation	1	−0.131	N/A^a^	N/A
	Significance (2-tailed test）	N/A	0.833	N/A	N/A
	Case number	5	5	N/A	N/A
**5-year survival rate (top 25%)**					
	Pearson correlation	−0.131	1	N/A	N/A
	Significance (2-tailed test)	0.833	N/A	N/A	N/A
	Case number	5	5	N/A	N/A
**Success number (bottom 25%)**					
	Pearson correlation	N/A	N/A	1	−0.945^b^
	Significance（2-tailed test	N/A	N/A	N/A	0.015
	Case number	N/A	N/A	5	5
**5-year survival rate (bottom 25%)**					
	Pearson correlation	N/A	N/A	−0.945^b^	1
	Significance (2-tailed test)	N/A	N/A	0.015	N/A
	Case number	N/A	N/A	5	5

^a^N/A: not applicable.

^b^Correlation is significant at the 0.05 level (2-tailed).

## Discussion

### Principal Findings

The purpose of this project was to investigate the relationship between GoFundMe cancer campaign features and the success of these campaigns and to understand the impact of text features on campaign success for various cancers. The results suggest that there are numerous interrelated factors that may contribute to the success of a cancer crowdfunding campaign and they can be categorized as campaign features and text features.

### Campaign Features

The number of donations to date, frequency of updates, sponsor of the fundraiser being a charity organization rather than an individual, and type of cancer were important contributing factors to the success of the fundraising project. On the basis of the number of donations, we specifically noted a Matthew effect, in that “For to all those who have, more will be given” [32].

Specifically, the more donations a project receives, the more people are drawn to donating money. Consistent with previous studies, people appeared to trust a project after viewing an expansive donation record, which may motivate potential donors to donate. Simultaneously, web-based medical crowdfunding campaigns usually receive a number of small donations; the higher the number of donors, the more likely a project is to meet its goal [33]. A reasonable and not too high goal amount is more conducive to fundraising success. This pattern may arise because excessively high fundraising goals are more difficult to achieve. Donors may feel more confident that smaller fundraising campaigns can be successful, incentivizing donor behavior, and increasing the likelihood of project success. Findings regarding the curvilinear relationship between funding targets and campaign success can aid future organizers in setting an appropriate funding target [34,35].

Campaigns by nonprofit organizations or institutions were more likely to achieve fundraising success than individual campaigns. This is consistent with other researchers’ findings, which show that organizations usually have greater social influence and credibility than individual organizers, thereby attracting more attention and trust [36,37]. Moreover, organization-based campaigns mostly involve large-scale charity events, which are more likely to arouse empathy and drive donations [38].

Campaign updates posted by organizers significantly and positively affect a project’s success, and genuine comments from others can boost a project’s credibility and attract potential donors [34]. The number of project updates indicates the importance of organizers attaching themselves to their campaigns. By posting updates, organizers can describe the beneficiary’s treatment status and use of funds. This information further improves a project’s transparency and credibility while enhancing readers’ sense of connection with the beneficiary, all of which foster fundraising success [39].

Organizers who emphasize the rarity of a beneficiary’s cancer type are more likely to receive donations. With GoFundMe, users are less likely to feel empathy when reading projects devoted to common types of cancer [11]. Donors are more likely to empathize with beneficiaries who have less (vs more) common types of cancer. This phenomenon is tied to the characteristics of a *warm glow* [11]; for our purposes, *warm glow* indicates whether donors express empathy when encountering crowdfunding campaigns. Overall, as presented in Table 13, cancer types with the highest fundraising success in our data set had greater overlap with the Centers for Disease Control and Prevention’s list of the 10 cancers with the highest mortality rates in the United States between 2014 and 2018. Cancers with high mortality rates and rarity were thus more likely to attract attention, elicit empathy, and promote donation behavior.

We speculate that crowdfunding success for cancers is not entirely contingent on the project description but is rather related to factors such as the 5-year survival rate, cure difficulty, and prevalence of the target cancer. Although lung cancer remains one of the deadliest cancers, developments in medicine have gradually reduced mortality rates for common cancers, such as cervical cancer and lung cancer, and this may be reflected in poorer fundraising outcomes. For example, estimates from the American Cancer Society have shown that the incidence and mortality associated with late-stage lung cancer have declined significantly in recent years [41]. This reduction is partially attributable to ongoing antismoking campaigns conducted by the Centers for Disease Control and Prevention. Because smoking, a personal behavior, is a key culprit in lung cancer, donors may be less likely to empathize with crowdfunding beneficiaries diagnosed with this type of cancer. In general, people are somewhat less willing to donate to campaigns for cancers, partly because of voluntary behavior [42]. In addition, timely diagnosis and rapid advances in lung cancer–related fields have decreased lung cancer mortality, providing a more promising outlook. In contrast, cancers of the bile duct and pancreas remain difficult to detect and diagnose in their early stages and have low survival rates in the middle and late stages. The late 5-year survival rate for bile duct cancer is less than half that for lung cancer. Mansour et al [43] reported that hilar cholangiocarcinoma, a type of bile duct cancer, is an uncommon malignant disease of the gastrointestinal tract [43].

**Table 13:** Top 10 cancers by rates of cancer deaths all types of cancer, United States, 2014 to 2018 and top 10 cancers by average percent funded.

Top 10 cancers by rates of cancer deaths	Age-standardized (rate)^a^	Top 10 cancers by percent funded
Lung and bronchus	38.5	Bile duct
Female breast	20.1	Pancreatic cancer
Prostate	19	Lymphoma
Colon and rectum	13.7	Brain tumor
Pancreas	11	Esophageal cancer
Liver and intrahepatic bile duct	6.6	Melanoma
Ovary	6.3	Prostate cancer
Leukemias	5.4	Leukemia
Non-Hodgkin lymphoma	4.4	Breast cancer
Corpus and uterus, NOS^b^	4.3	Colorectal cancer

^a^Rate per 100,000 people Source: US Cancer Statistics Working Group. US Cancer Statistics Data Visualizations Tool, based on 2020 submission data (1999-2018): US Department of Health and Human Services, Centers for Disease Control and Prevention, and National Cancer Institute [40], released in June 2021.

^b^NOS: not otherwise specified.

### Gender Differences

Women are typically thought to possess an advantage in the donation process [14]. However, our regression results revealed that words related to the beneficiary being woman were negatively associated with campaign success, that is, women had no advantage—and could even be at a disadvantage—in medical crowdfunding. This may be because most cancers typically linked to women are more common and easily curable, such as cervical cancer. The 5-year survival rate for cervical cancer exceeds 90% in the United States. Combined with the 5-year survival rates published in the National Cancer Institute’s Cancer Statistics Review [44], some of the least lethal cancers (lower than 25%) are prevalent in women in Table 14. Examples include thyroid cancer (98% of women) and cervical cancer (75% of women), both of which have higher 5-year survival rates. As a result, female-linked cancer campaigns have more difficulty raising money in cancer crowdfunding than male-linked cancer campaigns.

**Table 14:** Five-year survival rate and gender distribution for selected cancers.

Cancer type		5-year survival rate (%)	Gender distribution^a^
**Top 25%**			
	Bile duct	19	0
	Brain tumor	35	0
	Lymphoma	87	0
	Pancreatic cancer	9	0
	Melanoma	92	0
**Bottom 25%**			
	Thyroid cancer	98	1
	Bladder cancer	77	0
	Kidney cancer	75	0
	Cervical cancer	66	1
	Lung cancer	19	0

^a^Female cancers were coded as 1 and male cancers were coded as 0.

### Text Characteristics

Both longer project titles and descriptions, offering more detailed information, stimulated readers’ willingness to donate. Social psychology research suggests that increasing the number of arguments on a page can increase its persuasive impact power [45]. The overall average percent funded increased with the length of the project description when the text length was between 1 and 276 words. However, as the length of the text increased beyond 276 words, fundraising success decreased. An overly long text can increase a reader’s cognitive load and lead them to feel “manipulated.” Longer texts are also more likely to contain spelling and grammatical errors than shorter texts, limiting the effectiveness of the descriptions. Indeed, we found that a campaign’s number of spelling errors adversely affects fundraising results. This finding is consistent with other authors’ results. Fundraiser content with greater richness, correctness, and readability (perhaps reflecting better verbal and narrative skills) is more likely to capture the attention of donors, leading to a higher likelihood of receiving a donation [21,46]. Some fundraising founders turn to professionals to prepare textual materials to improve the quality of the project’s description [34,47]. Greenberg et al [48] suggested that the number of spelling errors is viewed as a lack of correctness by the public, whereas measures of readability (such as the Flesch-Kincaid grade level) indicate the education level needed for reading campaign materials. In addition, Mollick [49] concluded that spelling errors decreased the likelihood of fundraising success, whereas the number of Facebook friends, videos, and founder updates were all positively associated with funding success. Thus, while the effect of spelling mistakes may not be as strong as other textual characteristics, it is evident that the number of spelling errors in a project has an impact on fundraising results. Therefore, maintaining a moderate text length is best for crowdfunding results.

Text sentiment is an important factor in campaign success. Texts that contain positive emotions are more advantageous than those that contain negative emotions. Donors are more likely to be motivated by positive emotions, for example, “thank you,” “need your help,” and “please pray for.” These words imply an optimistic attitude of the fundraiser despite the illness and resonate with donors. Evidently, positive words create a more positive mood in readers; these fundraisers generate greater empathy among readers when browsing and underscore the value of donors’ contributions. In contrast, descriptions of beneficiaries’ current plight, expressing negative emotions (eg, fear, discontent, and resentment), or negative words (eg, negative terms and anxiety-related terms, tentative words, and risk-related words) in the project description were associated with lower levels of fundraising success. Thus, recommendations for campaign organizers are to display more positive emotions and use declarative words (eg, positive emotions; words related to certainty and leisure) to create a relaxed and optimistic atmosphere. Carefully crafted descriptions can inspire readers’ sympathy and ultimately increase the likelihood of donation.

More detailed emotional categories obtained through LIWC highlighted the varying influences of different emotional expressions on the success ratio. Campaigns with more positive emotion words, friend-related words, and leisure words were more likely to raise money. These words suggest more positive life attitudes, stronger social connections, and a more hopeful outlook on an individual’s future, generating greater empathy among readers and underscoring the value of donors’ contributions. In addition, project descriptions displaying concrete attitudes and determination (ie, certainty words) were more likely to be related to campaign success than text featuring uncertain words (ie, tentative words).

Finally, disparities were observed in the effects of emotional words on gender-specific cancer projects. Campaigns for male-linked cancers were more likely to be affected by the emotion generated from sadness words and anxiety words. Because of the more difficult cure and lower 5-year survival rates for cancers prevalent among men, the painful negative emotions portrayed in these words were more likely to garner sympathy from donors. Therefore, the emotions of helplessness and grief displayed in the descriptions of male cancer projects are more likely to result in effective fundraising.

### Limitations and Strengths

Although crowdfunding websites such as GoFundMe offer an opportunity to observe a wide range of thoughts and behaviors, our data have some limitations. First, people who earn a greater income and are fully insured might not need money from donations for cancer treatment. Therefore, they may not have created campaigns on crowdfunding websites. Second, the data included only US-based samples. It is possible that individuals in countries with universal health systems (eg, Canada) may not experience the same financial burden for cancer treatments. They may also describe their medical campaigns in a different manner than people in the United States, leading to different predictors of campaign success. In addition, we did not analyze the cultural background of the individuals requesting funds through crowdfunding nor did we assess donors’ cultural backgrounds. Cultural differences within the United States and between the United States and non-Western countries may limit the generalizability of these findings to donors’ perceptions and behaviors outside specific groups in the United States. Third, without a controlled experiment, we were unable to draw a causal relationship between campaign characteristics and fundraising outcomes. Nevertheless, our analyses identify likely candidate predictors of fundraising success in the United States. Finally, similar to the study by Silver et al [16], we were unable to obtain cancer stage information, which might be a significant variable contributing to crowdfunding success.

Despite these limitations, to the best of our knowledge, our study is the first cross-sectional study to provide large-scale quantitative support for the notion that difficult-to-treat cancers and high-mortality cancers receive more funds than common cancers and to analyze gender differences in the effects of emotional words in the text on the amount of funds raised.

### Future Research

Four areas for future research arise in this study. First, future studies are needed to replicate this analysis in other countries, especially those with different cultural norms and medical systems, to identify macrolevel influences on crowdfunding success. Second, future researchers could use more direct data collection methods to gather founders’ and donors’ demographic and socioeconomic data. With these data, more robust models to control for unobserved characteristics that may be correlated with crowdfunding success, such as the founder’s socioeconomic status, may help gain clues about additional drivers of crowdfunding success. Third, further studies should explore donation behavior using experimental settings to provide stronger evidence for a causal relationship between the characteristics of the funders/campaign descriptions and the donations they receive. Finally, future researchers could analyze the effects of visual and textual fusion on crowdfunding success.

### Conclusions

The effectiveness of medical crowdfunding for patients with cancer depends on a variety of interlocking factors. The most salient features are campaign features and campaign textual features. First, the type of cancer plays a role in funding success with rare cancers and cancers with lower survival rates (eg, bile duct cancer), easily outranking more common and less lethal cancers (eg, lung cancer). Second, men are more likely to receive higher funding rates than women. Indeed, a man with prostate cancer can raise up to 5.3 times more money than a woman with breast cancer.

However, the strongest predictor of success appears to be the goal amount (moderate vs too high). Setting a reasonable and not-too-high fundraising goal will greatly contribute to the success of a crowdfunding campaign. This is followed by the number of donations already made and the existence of comments from friends or donors.

Finally, the textual features that increase the likelihood of reaching one’s fundraising goal include moderately long texts, positive affect words, friend-related and leisure words (for select cancer types), words about cognitive insights, and visual sensory words, but, surprisingly, anger words and anxiety words for some cancers. In contrast, words that reduce the likelihood of campaign success include words about discrepancies, differentiation, words referring to women, health words, words related to risk, money, and religion, and negative emotion words. Taken together, the results of this study suggest that an upbeat campaign with many comments and early donations that projects optimism and personal insights without emphasizing religious or gender-related themes may be most likely to be successful.

## Abbreviations

CV: cross-validation
GloVe: Global Vectors for Word Representation
LIWC: Linguistic Inquiry and Word Count
PLR: penalized logistic regression
TF-IDF: term frequency–inverse document frequency
